# Reducing re-excision rates in breast conserving surgery with Margin Probe: systematic review

**DOI:** 10.1093/bjs/znad335

**Published:** 2023-11-22

**Authors:** Chara Rossou, Georgios Alampritis, Bijendra Patel

**Affiliations:** Barts Cancer Institute, Queen Mary University of London, Charterhouse Square, London, EC1M 6BQ, UK; Barts Cancer Institute, Queen Mary University of London, Charterhouse Square, London, EC1M 6BQ, UK; Barts Cancer Institute, Queen Mary University of London, Charterhouse Square, London, EC1M 6BQ, UK

## Abstract

**Introduction:**

Different intraoperative techniques with varying levels of evidence are available to decrease positive surgical margins during breast conserving surgery. The aim of this review is to assess the effectiveness of the MarginProbe® device as an intraoperative adjunct tool in reducing positive surgical margins, and subsequently exploring the effect on patient re-excision rates.

**Methodology:**

A systematic review of the available medical literature was conducted from 2007 to March 2022. A literature search of Cochrane, PubMed and Embase by two independent reviewers reviwers was performed to identify eligible articles looking at the primary outcome of percentage reduction in patient re-excision rates using MarginProbe®. Secondary outcomes analysed were comparison of tissue volume removed, absolute and relative reduction in re-excision rate, cosmetic outcome, as well as MarginProbe® sensitivity and specificity.

**Results:**

A total of 12 full text articles were identified. An independent samples *t*-test using a total of 2680 patients found a 54.68 per cent reduction in re-excision rate with the use of MarginProbe®, which was statistically significant with a large effect size (*P* < 0.001; *d* = 1.826). Secondary outcomes showed a relatively higher sensitivity of the MarginProbe® device, at the expense of decreased specificity, and no significant impact on cosmesis and volume of breast tissue excised.

**Conclusion:**

MarginProbe® is an effective intraoperative adjunct in breast-conservation surgery that reduces patient re-excision rates, with no adverse effects relating to breast cosmesis or increase in volume of excised tissue.

## Teaser

Different intraoperative techniques with varying levels of evidence are available for use during breast-conserving surgery, in an effort to decrease positive surgical margins and local recurrence. The aim of this systematic review was to show that the MarginProbe device can be used as an efficient intraoperative adjunct tool, to significantly reduce positive surgical margins and consequently patient re-excision rates. Our findings showed re-excision rates decreased by 54.68 per cent with use of the MarginProbe, with no adverse effects relating to breast cosmesis or increase in volume of breast tissue excised.

## Introduction

Breast cancer is reported to be responsible for 15 per cent of all newly diagnosed cases of cancer in the UK. As of March 2022, it is the most common cancer in the female population of the UK^1^ and the most common cancer worldwide^2^, making it a very relevant topic for research, especially as the incidence of breast cancer has been increasing^3^. Common breast cancer diagnoses are ductal carcinoma *in situ* (DCIS) and invasive breast cancer (IBC). The main treatment is surgery, which is often followed by radiotherapy and in some cases by endocrine and other systemic treatments (2022)^4,5^. Breast-conserving surgery (BCS) is preferred over mastectomy, due to improved cosmetic outcome^6^ and higher patient satisfaction. For patients diagnosed with early-stage breast cancer, BCS followed by adjuvant radiotherapy is the usual standard of care (2022)^7–10^. A challenge to this is that >20 per cent of patients undergoing BCS end up with positive surgical margins^8,11^ and therefore require re-excision surgery^10,12,13^. Besides the intraoperative MarginProbe**®** tool, other options are also available to surgeons, such as intraoperative ultrasound or gross examination. These options, however, have varying levels of evidence^14^.

The MarginProbe® System (Dilon Technologies, USA)^15^ is a relatively new device (2022) shown to have 70–100 per cent sensitivity and 70–87 per cent specificity^8,13,16^. It uses radiofrequency spectroscopy to identify cancerous tissue. This allows surgeons to identify positive surgical margins intraoperatively during BCS, and excise additional tissue where needed. This reduces subsequent re-excision surgeries by more than 50 per cent^8,16–19^. A big benefit is that additional excisions do not appear to lead to any removal of clinically significant additional breast tissue^20^. As with all new devices implemented in healthcare, the availability and cost of incorporating MarginProbe**®** is an important aspect, as is the training of relevant healthcare professionals on how to use the tool. It is also important to note that different policies regarding definition of an adequate margin also impact re-excision rates. The 2013 Society of Surgical Oncology/American Society for Radiation Oncology (SSO/ASTRO) guidelines defined free margins as ‘no ink on tumour’, meaning absence of cancerous cells next to an inked surface or edge of the specimen^21^. After these guidelines were adapted as the standard for adequate margins in invasive breast cancer, re-excision rates decreased significantly^22^. These guidelines are important to consider when comparing the published figures on MarginProbe**®** and re-excision rates.

A multicentre single-arm study compared the historical rates of re-excision surgery (39 per cent) to MarginProbe®, and showed a 56 per cent reduction in re-excision rates^23^. Procedural success improved by three times compared to standard of care alone^23^. Another single-centre retrospective review found that the rate of re-excision surgery for lumpectomy was 18.2 per cent compared to 9.2 per cent for the group not using *versus* using the device for positive margin detection, respectively^19^. A prospective phase II clinical trial, however, advocated that the device leads to a very small and negligible reduction of 2 per cent in re-excision rates^24^. Moreover, the same study claims that MarginProbe® may not be especially useful if the surgeon performing BCS already has a history of low re-excision rates^24^.

The aim of the systematic review is to assess the effectiveness of the MarginProbe® device as an intraoperative adjunct tool in reducing positive surgical margins, and subsequently exploring the effect on re-excision rates.

## Methods

The systematic review was prospectively registered on PROSPERO on 11 March 2022 with registration number CRD42022296889.

### Data collection and sources

Data collection was conducted from secondary sources and online search engines and databases. Appropriate PRISMA guidelines were followed. PubMed, Cochrane CENTRAL and Embase were searched for the relevant studies that included MarginProbe® in the context of BCS from the year 2007. The full search strategy can be found in the *Supplementary material S1*. No limits were applied to these searches. The data collection period lasted from October 2021 to March 2022. Consequently, full-text articles were manually assessed and 12 articles in total were found to be eligible for the systematic review.

### Eligibility criteria

Inclusion criteria included women over 18 years of age with either non-palpable DCIS or IBC. All patients were eligible and had opted for BCS, whether they had received previous neoadjuvant chemotherapy (NAC) treatment or not. Therefore, both ‘NAC + BCS’ and ‘BCS only’ patient groups were included during article selection.

Exclusion criteria were patients with multicentric or bilateral disease, diffuse microcalcifications, patients with a history of surgery in the ipsilateral breast, prior radiotherapy, pregnancy, and lactation. Articles including another method of intraoperative treatment with MarginProbe® were excluded. Untranslatable articles in a language other than English were excluded.

### Extracted data

Data extracted from the eligible articles included: type of breast cancer, prior NAC treatment, re-excision percent in control group, re-excision percent in intervention group, relative percent reduction in re-excision rate, absolute percent reduction in re-excision rate, tissue volume removed, sensitivity and specificity of the MarginProbe® device, breast cosmesis outcomes, and lastly, number of patients eligible for statistical analysis.

### Risk of bias

Risk of bias and quality assessment of studies were performed before statistical analysis commenced. The revised RoB-2 Cochrane tool was used to assess risk of bias in the four eligible RCT. Cochrane ReviewManager Web was used and edited online (*Figs S1*, *S2*). The ROBINS-I tool^25^ was used to assess the eight non-randomized studies (*Table S1*).

### Statistical methods and analyses

Primary outcome measures analysed in the eligible studies: reduction in re-excision rates attributed to intraoperative MarginProbe® use (12/12 studies). Only the primary outcome measure had enough data in the eligible articles to be suitable for statistical analysis. Secondary outcome measures analysed in the eligible studies were comparison of tissue volume removed between the control and intervention groups (7/12 studies), absolute reduction in re-excision rate (12/12 studies), relative reduction in re-excision rate (12/12 studies), breast cosmetic outcome comparison between the control and intervention groups (3/12 studies), MarginProbe® sensitivity (3/12 studies), MarginProbe® specificity (3/12 studies).

A Shapiro–Wilk test showed that the data were normally distributed. An independent samples *t*-test was then conducted, in order to compare the differences in re-excision rates between control and intervention groups, with the aim to conclude if there is a significant reduction in the re-excision rate. No missing data were imputed. For the primary outcome, statistics were performed using *α* =0.05 significance level.

## Results

### Literature search and study selection

**Fig. 1:**
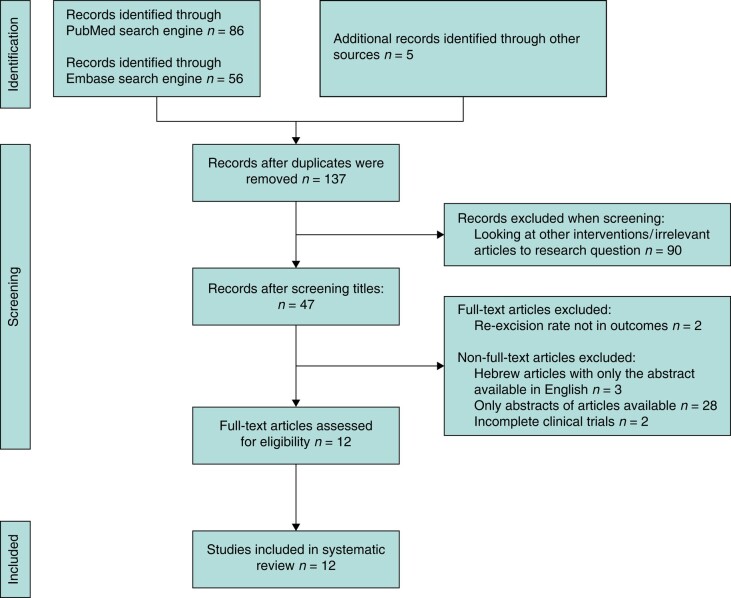
PRISMA flow diagram of selected studies

A total of 12 studies satisfied the eligibility criteria for the systematic review. *Figure 1* provides a flow diagram of how articles were screened and excluded. Duplicates were manually removed and checked via Rayyan software. All 12 studies have the same primary outcome and aim to compare the percentage of re-excision surgeries with the use of MarginProbe® during BCS, *versus* the percentage of re-excision surgeries when the device was not used during BCS. Also, most of the eligible studies report on a proportion of the secondary outcomes. *Table 1* summarizes the characteristics of included studies. There is heterogeneity in terms of quality of the 12 studies included, as they have varying study designs and bias assessment outcomes.

**Table 1:** Patient characteristics of all included studies

Author	Patients eligible for statistical analysis	Prior NACT treatment	Type of breast cancer
Elyse LeeVan *et al*.	60	No	DCIS, IBC
Tanir M. Allweis *et al*.	293	No	DCIS, IBC
Marc Thill *et al*. (2011)	22	No	DCIS
Ronald J. Rivera *et al*.	596	Unclear	DCIS, IBC
Marc Thill *et al*. (2013)	42	No	DCIS
Freya Schnabel *et al*.	596	No	DCIS, IBC
Molly Sebastian *et al*.	165	Unclear	DCIS, IBC
Jens-Uwe Blohmer *et al*.	322	Yes	DCIS, IBC
Jeffrey Coble *et al*.	256	Unclear	DCIS, IBC
Amanda Kupstas *et al*.	240	No	DCIS, IBC
Rula C. Geha *et al*.	46	No	DCIS, IBC
Cindy Cen *et al*.	42	Yes	DCIS, IBC
Total number of articles: **12**	Total patient number analysed: **2680**	Seven articles exclude NACT + BCS patients	Ten articles include DCIS ± IBC patients
Four randomized controlled trials		Two articles include NACT + BCS patients	Two articles only include DCIS patients
Eight non-randomized trials		Three articles are unclear about including NACT + BCS patients	

Abbreviations DCIS = ductal carcinoma *in situ*; IBC = invasive breast cancer; NACT = neoadjuvant chemotherapy.

### Risk of bias assessment findings

Risk of bias was assessed for the selected RCTs using the Cochrane RoB-2 tool, and found three articles with low risk of bias and one article with high risk (*Figs S1*, *S2*). Risk of bias was assessed for the non-randomized studies using the ROBINS-I tool and found four articles with low risk of bias and four articles with moderate risk of bias, as shown in *Table S1*.

### Characteristics of included studies

Overall, from the 12 eligible articles, a total of 2680 patients were analysed statistically. From the pool of eligible articles, four were RCTs of higher-quality evidence and the remaining eight were non-randomized trials. Only two of these articles (*n* = 364) include the ‘NAC + BCS’ patient group.

A mean (s.d.) of 54.68 per cent (18.78) was calculated for relative reduction in re-excision, with a mean of 25.80 per cent 10.12) for control group re-excision rate and a mean of 10.93 per cent (5.49) for the intervention group re-excision. A mean of 69.07 per cent was found for device sensitivity and a lower mean of 63.47 per cent for device specificity. In terms of tissue volume removed, four articles (*n* = 1474) reported very similar volumes, one article reported no difference and two articles found a decreased volume of tissue excised. Breast cosmesis was comparable between control and intervention groups in two articles (*n* = 889) and was termed acceptable in one article (*Table 2*).

**Table 2:** MarginProbe findings regarding primary and secondary outcomes

	Control group re-excision, %	Intervention group re-excision, %	Relative reduction, %	Absolute reduction, %	Sensitivity, %	Specificity, %	Tissue volume removed	Cosmesis
Elyse LeeVan *et al*.	8.60	6.60	23.00	2.00	75.00	94.00	n/a	n/a
Tanir M. Allweis *et al*.	12.70	5.60	56.00	7.10	n/a	n/a	No difference	Comparable in device/control
Marc Thill *et al*. (2011)	38.80	18.00	53.60	20.80	n/a	n/a	n/a	n/a
Ronald J. Rivera *et al*.	29.90	14.10	57.00	15.80	n/a	n/a	Very similar	Comparable in device/control
Marc Thill *et al*. (2013)	39.00	17.00	56.00	22.00	57.00	50.00	Very similar	Acceptable
Freya Schnabel *et al*.	25.80	19.80	23.00	6.00	75.20	46.40	Very similar	n/a
Molly Sebastian *et al*.	25.80	9.70	61.00	16.10	n/a	n/a	n/a	n/a
Jens-Uwe Blohmer *et al*.	29.70	14.60	51.00	15.10	n/a	n/a	n/a	n/a
Jeffrey Coble *et al*.	15.10	6.60	57.00	8.50	n/a	n/a	Decreased	n/a
Amanda Kupstas *et al*.	18.20	9.20	50.00	9.00	n/a	n/a	Very similar	n/a
Rula C. Geha *et al*.	35.00	4.00	88.00	31.00	n/a	n/a	Decreased	n/a
Cindy Cen *et al*.	31.00	6.00	80.60	25.00	n/a	n/a	n/a	n/a
								
Mean (SD), %	25.80 (10.12)	10.93 (5.49)	54.68 (18.78)	14.87 (8.69)	69.07 (10.45)	63.47 (26.50)	Four articles found very similar volumes removed	Two articles found breast cosmetic outcome comparable between the device and control groups
Minimum-Maximum	8.60–39.00	4.00–19.80	23.00–88.00	2.00–31.00	57.00–75.20	46.40–94.00	One article found no difference in volume removed Two articles found decreased volume removed	One article found breast cosmetic outcome acceptable between the device and control groups

### Re-excision rates

The mean (s.d.) re-excision rate was 10.93 per cent (5.49 per cent) for the ‘Re-excision rate in intervention group with MarginProbe® use’. The mean (s.d.) re-excision rate was 25.80 per cent (10.12 per cent) for the ‘Re-excision rate in control group without MarginProbe use’. The re-excision rate of the intervention group was significantly reduced compared to the control group without MarginProbe® use; *t*(22) = 4.473, *P* = 0.001, effect size *d* = 1.826. Descriptive and inferential statistics are shown in *Table 3*.

**Table 3:** Descriptive and inferential statistics for re-excision rate

	Control group re-excision rate without MarginProbe, %	Intervention group re-excision rate with MarginProbe, %
Mean	25.80	10.93
95% C.I.	(19.37–32.23)	(7.44–14.42)
Standard deviation	10.12	5.49
Standard error mean	2.92	1.59

	Control group without MarginProbe use *versus* Intervention group with MarginProbe use	
*t*-Statistic	4.473	
df	22.000	
*P*	<0.001*	
Cohen's d	1.826	

^*^ *P* < 0.05 indicates statistical significance.

## Discussion

The aim of this systematic review was to establish how effective the use of the intraoperative MarginProbe® device is during breast-conservation surgery, in terms of reducing re-excision rates in breast cancer patients. A significant reduction of re-excision rates was shown when comparing the intervention group with the use of MarginProbe® and the control group without the use of the device in BCS.

This systematic review brings together the results of the 12 eligible articles chosen according to the eligibility criteria, with a total sample size of 2680 patients. The four RCTs included were of a higher level of evidence than the eight non-randomized trials. The eligible studies were chosen from a pool of studies not markedly large in number, reflecting the need for more research on the topic area. The studies that were analysed differ in risk of bias and study design. The observed heterogeneity further emphasizes the need to study the MarginProbe® device in depth, and to further elucidate whether indeed this tool is effective and should be used as a tool during BCS. Variability of evidence in the current literature shows a lack of clarity within the current state of understanding and knowledge on this relatively new device, and calls for a much-needed, better-informed future consensus on its efficiency.

The results of this systematic review could have significant implications on practice. The analysis found a relatively large mean relative reduction in re-excision rate of 54.68 per cent (±18.78) overall from the 12 included articles, and it can therefore be concluded that MarginProbe® indeed seems to reduce re-excision rates by at least half, which is in itself a very important clinical outcome. Most data published on this device agree with the conclusion that it reduces subsequent surgeries by 50 per cent or more^8,16–19^. A single-centre RCT found a very large relative reduction in re-excision rate of 88 per cent, and an absolute reduction of 31 per cent between the intervention and control groups^13^. This study looked at device use in both IBC and DCIS^13^, and of the 12 eligible studies included in this review, it found not only a >50 per cent reduction, but also the largest relative reduction in re-excision rates. Another study looking at the ‘NAC + BCS’ patient group only reports a very large relative reduction of 80.60 per cent^26^. Most of the trials on MarginProbe® exclude the NAC + BCS patient group, so this result suggests a potentially wider applicability of the device. However, more studies are needed on the NAC + BCS patient group in order to draw reliable conclusions. The multicentre Pivotal Trial also found a relative reduction in re-excision rate of 57 per cent, thereby reducing reoperations by more than half with an absolute reduction in re-excision rate of 15.8 per cent^18^. A multicentre RCT enrolling 596 patients also found a relative reduction in reoperation rate of 23 per cent, which was less than the advocated 50 per cent reduction. The authors still encourage adjunctive use of the device in institutions where routine intraoperative pathology assessment is not available^16^. Overall, most articles on MarginProbe® device performance encourage use in BCS patients, claiming that it is highly efficient in reducing patient reoperations and consequently reducing the burden of reoperations on the healthcare system and on the patients themselves.

A prospective phase II trial that found a negligible absolute reduction in re-excision rate of 2 per cent, with a low relative reduction rate of re-excisions at 23 per cent, claims that MarginProbe® has the same performance as gross pathological examination and specimen radiography^24^. The trial correctly states it is worth taking into account baseline re-excision rates in centres using other margin assessment methods, such as intraoperative specimen X-ray, because if the adopted methods already have low baseline re-excision rates, then the adjunctive use of MarginProbe® device to the standard of care will probably lead to a negligible reduction in re-excision rate^24^. There is certainly a cost for the device to be implemented in any institution, although this is usually balanced out by the reduction in reoperations^8,16^. At the same time, this prospective trial included a small sample size of 60 patients and the experience of only a single surgeon, which could impact the reliability of results.

Secondary outcomes were extracted from the 12 eligible articles that included information on these, but there were not enough data to undergo statistical testing. However, some trends could be observed, as stated in the articles. BCS is an equally effective alternative option to mastectomy for patients with early-stage breast cancer. Because BCS allows most of the breast tissue to be preserved, factors such as cosmesis and amount of tissue volume excised are of importance. Two articles (*n* = 889) claim that breast cosmesis was comparable^18,23,27^ between the control and intervention group. Also worthy of note were the results of the Pivotal Trial (*n* = 596) at 21 different institutions, where the volume of tissue removed with MarginProbe® during primary BCS was slightly higher in the intervention group, which would logically lead to worse cosmetic outcome^18^. However, as BCS with the MarginProbe® device led to a 57 per cent reduction in the need of re-excision surgeries, and after analysing the total tissue volume removed, the control and intervention group findings for breast cosmetic outcome were still very similar after normalization to bra cup sizes^18^. Therefore, the Pivotal Trial found that MarginProbe® could even allow for better cosmetic outcomes due to the overall smaller volumes of total tissue resected^18^.

Cosmetic outcome itself can also be affected by a variety of factors, such as the volume of tissue excised, size and location of the tumour in the breast, adjuvant radiotherapy, and the need for re-excision procedures^16^. Across the seven eligible studies that included data on tissue volume removed, the results were very comparable^19^ between control and intervention groups, or even slightly decreased^13,28^ in the intervention groups using MarginProbe®. From these articles, when taking into account cosmesis and volume of breast tissue excised, it can be reliably concluded that the device has little impact on the cosmetic outcome or none at all on the amount of breast tissue excised^16^.

Because the MarginProbe® system was designed for maximal detection of positive margins, there is an emphasis on the sensitivity of the device^16^. It therefore follows that this higher sensitivity would inevitably mean a lower specificity and an increase in false-positive results^16^. False-positive results mean a higher volume of tissue excised, which could be a big disadvantage leading to worse cosmesis. Important to note is that when using a clear margin threshold of 1 mm, sensitivity was found to be higher at 65 per cent while specificity remained the same at 50 per cent^23^. Evaluation of the device in the eligible articles showed a relatively higher sensitivity in detecting positive margin status. The lower specificity is seen as acceptable because the overall additional tissue excised and cosmetic outcome remain similar.

There is no established standard of care for intraoperative margin assessment and management for BCS^29^, even though there are many different methods available, of which the MarginProbe® is one. Others include intraoperative ultrasound, radioactive seed localization (RSL), radio-guided occult lesion localization (ROLL), gross examination, frozen section analysis, imprint cytology, cavity-shave margins and specimen radiography, which are all methods that have been used intraoperatively^14^ in the hopes of decreasing positive margins and achieving a better surgical outcome. RSL and ROLL are both alternative localization techniques for non-palpable tumours^30^, which decrease the rate of positive margins. High-resolution F-fluoro-deoxyglucose (FDG) PET and X-ray computed tomography have good sensitivity and specificity, and these can decrease re-excision rates^31^. The same applies to the use of ultrasound technique for palpable tumours. A multicentre RCT advocates a significant reduction of 9.9 per cent in margin involvement and excision volumes for ultrasound-guided BCS when compared to palpation-guided surgery. Ultrasound has not, however, shown a difference in recurrence rates and is not generally implemented in hospitals^32^. Intraoperative pathology techniques, if these are available for use, can also lower positive margin rates. In situations where such techniques are not available, the MarginProbe® device and cavity-shave margins seem to be good alternative options^14^. A multicentre RCT showed a decrease in re-excision rates of 14.8 per cent with cavity-shave margins *versus* non cavity-shave margins^33^. Another relatively new device called the Histolog® Scanner allows real-time confocal assessment of lumpectomy margins and can lead to a 75 per cent reduction of reoperation rates. However, these results need to be further confirmed in a future phase III trial^34^.

The strengths of this review include the comprehensive search to find all potentially eligible articles and the adequately large sample size found, despite the relatively few articles on the research topic area. Some of these articles were multicentre rather than single centre, making the results more reliable and potentially generalizable.

This review has methodological limitations due to the limitations in the available literature. Raw data were not available to use, so quality of data could be improved, and interpretation could be limited. There was no opportunity to independently assess ‘NAC + BCS’ patient outcomes due to the lack of available evidence. Another important, yet lacking, aspect in the literature is the different situations in which MarginProbe® performs, such as lobular cancer and DCIS, so sufficient conclusions cannot be made. Re-excision rate figures are thus generalized in all situations of BCS where MarginProbe® is used as an adjunct tool. In general, RCTs on MarginProbe are lacking in the literature and non-randomized trials are of a lower level of evidence. Several limitations are also present in the individual studies analysed. Some lacked a matching cohort of patients and collected data retrospectively from a historical patient cohort. Furthermore, some of the studies included small sample sizes and were single-centre or single-surgeon experiences. Patient selection could be an issue as a number of the eligible patients recruited were inevitably older and therefore had less-dense breasts and therefore lower rate of re-excision^35^. Lastly, some articles report re-excision rates for more than one margin width definition^17,23^, and even though increasing margins have not shown lower local recurrence so far^36^, there were not enough data to statistically analyse this.

Future work is needed to understand the relationship between the different margin distances that can be used and how this could affect local recurrence. Further research is needed on eligible patient groups such as NAC + BCS patients for which current evidence is lacking. If NAC + BCS patients have higher percentages of re-excision rates compared to patients receiving only primary BCS, then MarginProbe® could be particularly helpful in potentially mitigating these higher re-excision rates. Positive evidence in this context could be significant in terms of external validity for the device.

## Supplementary Material

znad335_Supplementary_Data

## Data Availability

The first author can provide access to data upon request
