# Supplementary material for: Reducing re-excision rates in breast conserving surgery with Margin Probe: systematic review
Source: Br J Surg. 2023 Nov 22;111(1):znad335. doi: 10.1093/bjs/znad335 (PMC10776367; doi:10.1093/bjs/znad335)
Supplement: znad335_Supplementary_Data [file znad335_supplementary_data.docx]

**Title:**Effectiveness of intraoperative margin assessment in breast

conserving surgery with MarginProbe® as a tool to reduce reexcision rates: Systematic Review

Authors:
Chara Rossou^1^, Georgios Alampritis^1^, Bijendra Patel^2^

^1^Affiliation: Barts Cancer Institute, Queen Mary University of London

^2^Affiliation : Professor of Surgery, Barts Cancer Institute, Queen Mary University of London

**Corresponding author:** Chara Rossou^1^, rossouchara@gmail.com

**ORCID ID**: 0009-0003-3012-8435

**Supplementary Materials - Index**

| **Supplementary Methods** |  |
| --- | --- |
| Methods S1.  In “Data Collection and Sources” section of Methods, the search strategy is mentioned to be given in the supplementary material. Search strategy and individual terms are given below. | *pag. 5* |
| Detail | *pag. Y* |
| **Supplementary Results** |  |
| Detail | *pag. X* |
| Detail | *pag. Y* |
| **Supplementary Appendixes** |  |
| Detail | *pag. X* |
| Detail | *pag. Y* |
| **Supplementary Figures and Tables** |  |
| Supplementary Figure S1.  In risk of bias assessment findings risk of bias is reported and then shown via the ‘Risk of bias graph for included RCTs (RoB-2)’ - given as Figure S2. | *pag. 6,9* |
| Supplementary Figure S2.  In risk of bias assessment findings risk of bias is reported and then shown via the ‘Risk of bias graph for included RCTs (RoB-2)’ - given as Figure S3. | *pag. 6,9* |
| Supplementary Table 1.  In risk of bias assessment findings risk of bias is reported and then shown via the ‘Risk of Bias table for included non-RCTs (ROBINS-I)’ – given as Table S4. | *pag.6, 9* |
| **References** | *pag. Z* |
|  |  |

**Supplementary Methods:**

**S1 - The full search strategy:**

**Term 1:** “re-excision” OR “re-excision rates” OR “re-excision rate” OR “re-excision rate reduction” OR “re-excision rates reduction” OR “re-excision reduced” OR “re-excision decrease” OR “re-excision surgery” OR “repeat excision” OR “reduced” OR “re excision” OR “re excision rates”

**Term 2:** “BCS” OR “Breast conserving surgery” OR “Breast conservation surgery” OR “breast conserving” OR “breast conservation” OR “breast cancer surgery” OR “breast surgery”

**Term 3**: “Intraoperative” OR “Intraoperative assessment” OR “Intra-operative” OR “Intra-operative assessment” OR “Intraoperative margin assessment” OR “real-time margin assessment” OR “real time margin” OR “intra-operative margin” OR “margins” OR “margin” OR “assessment” OR “intraoperative”

**Term 4:** “MarginProbe” OR “MarginProbe®” OR “MarginProbe® system” OR “MarginProbe® device” OR “MarginProbe® tool” OR “MarginProbe® adjunct” OR “Margin Probe device” OR “Margin Probe system” OR “Margin Probe tool” OR “Margin Probe adjunct” OR “Margin Probe” OR “Marginprobe” OR “MarginProbe device” OR “MarginProbe system” OR “radio-frequency spectroscopy” OR “radio frequency spectroscopy” OR “radio frequency” OR “radio frequency spectroscopy margin probe”

**Search Strategy:** ((((((((((((((re-excision) OR (re-excision rates)) OR (re-excision rate)) OR (re-excision rate reduction)) OR (re-excision rates reduction)) OR (re-excision reduced)) OR (re-excision decrease)) OR (re-excision surgery)) OR (repeat excision)) OR (reduced)) OR (re excision)) OR (re excision rates)) AND (((((((BCS) OR (Breast conserving surgery)) OR (Breast conservation surgery)) OR (breast conserving)) OR (breast conservation)) OR (breast cancer surgery)) OR (breast surgery))) AND ((((((((((((Intraoperative) OR (Intraoperative assessment)) OR (Intra-operative)) OR (Intra-operative assessment)) OR (Intraoperative margin assessment)) OR (real-time margin assessment)) OR (real time margin)) OR (intra-operative margin)) OR (margins)) OR (margin)) OR (assessment)) OR (intraoperative))) AND ((((((((((((((((((MarginProbe) OR (MarginProbe®)) OR (MarginProbe® system)) OR (MarginProbe® device)) OR (MarginProbe® tool)) OR (MarginProbe® adjunct)) OR (Margin Probe device)) OR (Margin Probe system)) OR (Margin Probe tool)) OR (Margin Probe adjunct)) OR (Margin Probe)) OR (Marginprobe)) OR (MarginProbe device)) OR (MarginProbe system)) OR (radio-frequency spectroscopy)) OR (radio frequency spectroscopy)) OR (radio frequency)) OR (radio frequency spectroscopy margin probe))

**Supplementary Results**

**Supplementary Appendixes**

**Supplementary Figures and Tables**

**
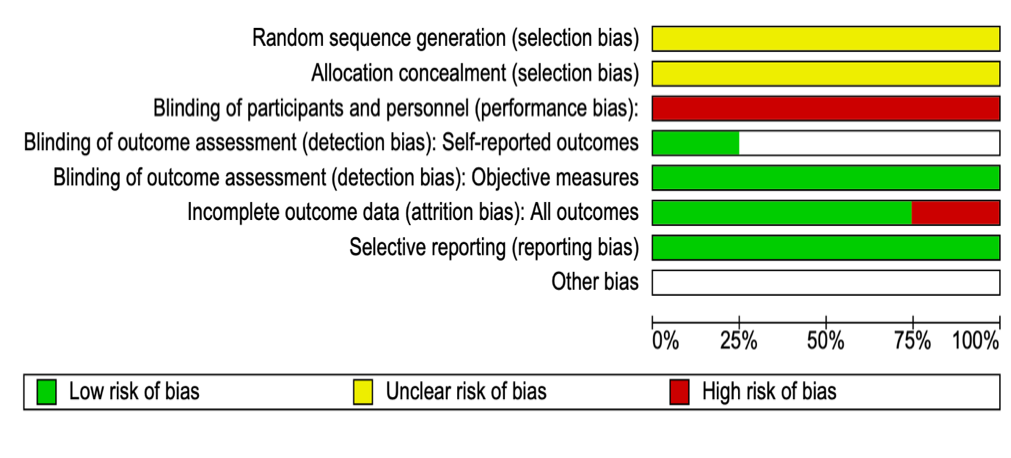
**


 **Figure S1:** Risk of bias graph for included RCTs (RoB-2)

**
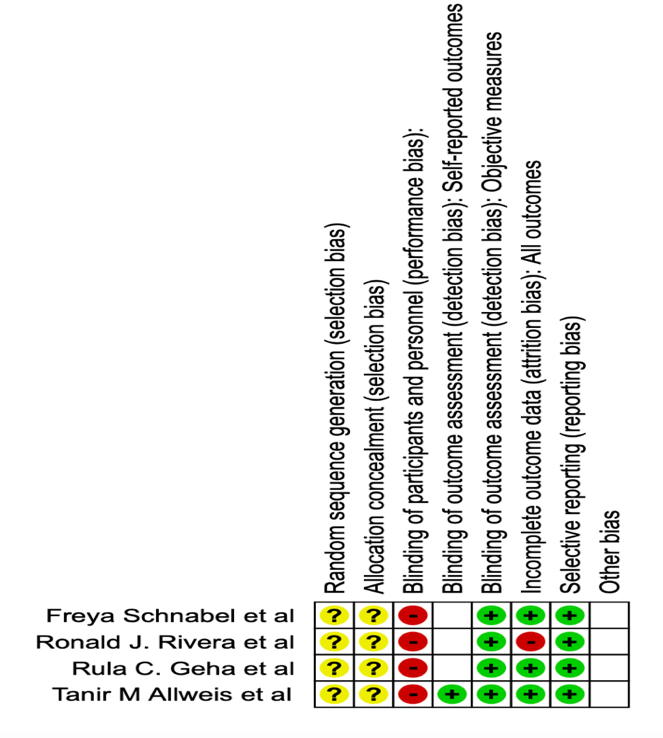
**

**Figure S2:** Risk of bias for included RCTs (RoB-2)

| **No** | **Author** | **Selection bias** | **Confounding bias** | **Classification of interventions bias** | **Deviations from intended interventions bias** | **Missing data bias** | **Measurement of outcomes bias** | **Selection of reported result bias** |
| --- | --- | --- | --- | --- | --- | --- | --- | --- |
| **1** | **Cindy Cen et al** | Low risk | Low risk | Low risk | Low risk | Low risk | Low risk | Low risk |
| **2** | **Elyse LeeVan  et al** | Moderate risk | High risk | Low risk | Low risk | Low risk | Unclear risk | Low risk |
| **3** | **Amanda Kupstas  et al** | Low risk | Low risk | Low risk | Low risk | Low risk | Unclear risk | Low risk |
| **4** | **Jeffrey Coble  et al** | Low risk | Low risk | Low risk | Low risk | Low risk | Low risk | Low risk |
| **5** | **Jens-Uwe Blohmer  et al** | Low risk | Low risk | Low risk | Low risk | Low risk | Low risk | Low risk |
| **6** | **Molly Sebastian et al** | Low risk | Low risk | Low risk | Low risk | Low risk | Low risk | Low risk |
| **7** | **Marc Thill et al  (2013)** | Low risk | Low risk | Low risk | Low risk | Low risk | Low risk | Low risk |
| **8** | **Marc Thill et al  (2011)** | Moderate risk | Low risk | Low risk | Low risk | Moderate risk | Unclear risk | Low risk |

**Table S1:** Risk of Bias table for included non-RCTs (ROBINS-I)

**References**
